# Annexin A2 binds to vimentin and contributes to porcine reproductive and respiratory syndrome virus multiplication

**DOI:** 10.1186/s13567-018-0571-5

**Published:** 2018-07-27

**Authors:** Xiao-Bo Chang, Yong-Qian Yang, Jia-Cong Gao, Kuan Zhao, Jin-Chao Guo, Chao Ye, Cheng-Gang Jiang, Zhi-Jun Tian, Xue-Hui Cai, Guang-Zhi Tong, Tong-Qing An

**Affiliations:** 1 State Key Laboratory of Veterinary Biotechnology, Harbin Veterinary Research Institute, Chinese Academy of Agricultural Sciences, Harbin, 150069 China; 2 Shanghai Veterinary Research Institute, Chinese Academy of Agricultural Sciences, Shanghai, 200241 China

## Abstract

Porcine reproductive and respiratory syndrome virus (PRRSV) is an important globally distributed and highly contagious pathogen that has restricted cell tropism in vivo and in vitro. In the present study, we found that annexin A2 (ANXA2) is upregulated expressed in porcine alveolar macrophages infected with PRRSV. Additionally, PRRSV replication was significantly suppressed after reducing ANXA2 expression in Marc-145 cells using siRNA. Bioinformatics analysis indicated that ANXA2 may be relevant to vimentin, a cellular cytoskeleton component that is thought to be involved in the infectivity and replication of PRRSV. Co-immunoprecipitation assays and confocal analysis confirmed that ANXA2 interacts with vimentin, with further experiments indicating that the B domain (109–174 aa) of ANXA2 contributes to this interaction. Importantly, neither ANXA2 nor vimentin alone could bind to PRRSV and only in the presence of ANXA2 could vimentin interact with the N protein of PRRSV. No binding to the GP2, GP3, GP5, nor M proteins of PRRSV was observed. In conclusion, ANXA2 can interact with vimentin and enhance PRRSV growth. This contributes to the regulation of PRRSV replication in infected cells and may have implications for the future antiviral strategies.

## Introduction

Porcine reproductive and respiratory syndrome (PRRS), caused by the PRRS virus (PRRSV), is one of the most economically important diseases affecting the global pig farming industry. It is characterized by late term gestation reproductive failure in sows and general respiratory symptoms in pigs of all ages and sexes [1–4]. PRRSV is a member of the *Nidovirales* order (*Arteriviridae* family) and consists of an enveloped 15 kb positive-strand RNA genome containing nine open reading frames (ORFs) [5]. ORF1a and ORF1b encode viral replicase polyproteins, while ORF2a, ORF2b, and ORFs 3–7, encode the viral structural proteins GP2, E, GP3, GP4, GP5, M, and N, respectively. All of these structural proteins are required for the PRRSV replication [6–8].

Annexin A2 (ANXA2) is a member of the annexin family of calcium-dependent proteins expressed in many cells [9], which share structural and functional features [10]. ANXA2 is also a multifunctional protein involved in many biological processes, including endocytosis, exocytosis, membrane domain organization, extracellular receptor activity, signal transduction, protein assembly, transcription and mRNA transport, as well as numerous pathologies [11–15]. For example, ANXA2 is involved in the production of classical swine fever virus infectious particles [16]. ANXA2 was identified as a novel host factor contributing to the formation of infectious HCV particles [17]. Vimentin, a cellular cytoskeleton component, also plays an important role in the infection process of PRRSV as the anti-vimentin mAb has been shown to block PRRSV infection [18, 19]. Vimentin is also thought to be involved in PRRSV replication and transportation of the virus into cells by forming a complex with other intermediate filament components [20]. However, the relationship between ANXA2 and vimentin is unknown.

In previous experiment, we examined PAMs infected with the highly pathogenic PRRSV HuN4 strain. We found that ANXA2 was differentially expressed in infected cells and bioinformatics analysis indicated that ANXA2 may associate with vimentin. To investigate the role of ANXA2 in PRRSV replication and the relationship of ANXA2 and vimentin, RNA interference assay showed that ANXA2 could promote PRRSV infection. In addition, we had confirmed that ANXA2 could interact with the vimentin for the first time, and ANXA2 together with vimentin, form a complex that could bind to PRRSV N protein. These findings contribute to understanding the cellular proteins on the role of regulating PRRSV replication and may have implications for the future control of this important disease.

## Materials and methods

### Cells and virus

PAMs were harvested from 4-week old PRRSV-negative piglets in sterilized phosphate-buffered saline (PBS) by alveolar lavage and then maintained in RPMI-1640 medium. The animal experiments in this study were conducted with recommendations in the Chinese Regulations of Laboratory Animals—The Guidelines for the Care of Laboratory Animals (Ministry of Science and Technology of People’s Republic of China) and Laboratory Animal-Requirements of Environment and Housing Facilities (GB14925-2010, National Laboratory Animal Standardization Technical Committee). Marc-145 cells and human embryonic kidney (HEK) 293T cells were grown in Dulbecco’s modified Eagle’s medium (DMEM), supplemented with 10% fetal bovine serum (FBS). PRRSV HuN4 (GenBank No. EF635006), a highly pathogenic PRRSV strain [21], was maintained in our laboratory.

### Two-dimensional difference gel electrophoresis (2D-DIGE) and image analysis

PAMs were infected with PRRSV HuN4 at a MOI of 0.01. At 48 hours post-infection (hpi), cells were harvested, treated, and separated by 2D-DIGE as previously described [22, 23]. Briefly, samples were actively rehydrated in 24 cm precast immobilized pH gradient (IPG) strips (GE Healthcare, Chicago, IL, USA) for 12 h using an Ettan IPGphor three system (GE Healthcare). Isoelectric focusing (IEF) was then performed for a total of 76 kV h. Once the IEF was completed, the IPG strips were equilibrated in equilibration buffer supplemented with 1% DTT for 15 min and washed for another 15 min. Separation in the second dimension was performed using 12.5% SDS-PAGE. After SDS-PAGE, gels were scanned on a Typhoon TRIO Scanner (GE Healthcare). Analysis of the 2D-DIGE was performed using DeCyder 6.5 software (GE Healthcare), according to the manufacturer’s recommendations.

### Identification by mass spectrometry and Western blotting

Differentially expressed protein spots of interest were manually excised, treated, and identified by mass spectrometry using protocols and methodology from a previous study [22]. The expression levels of ANXA2 in HuN4 infected PAMs at 48 hpi were determined via Western blotting using a commercial polyclonal rabbit anti-ANXA2 mAb (Cell Signaling Technology, Danvers, MA, USA).

### Bioinformatics analysis

STRING, an open source web-based tool consisting of established and predicted protein interactions, was utilized to analyze the protein–protein interaction networks, as described previously [24]. This tool integrates biomolecular interaction networks using high throughput expression results, and other molecular states, in a unified conceptual framework.

### Knockdown and overexpression of ANXA2

Small interfering RNAs (siRNA) targeting the ANXA2 gene (siANXA2) were synthesized by GenePharma (Shanghai, China). The siRNA sequences (5′-GGACAUUAUUUCGGAUACATT-3′) used have been previously reported and validated [25]. The non-target control siRNA (siNC) sequence (5′-UUCUCCGAACGUGUCACGUTT-3′) was confirmed to have no matches in either the viral or *Chlorocebus aethiops* (green monkey) genome. Marc-145 cells were transfected with either 100 nM siANXA2 or siNC using X-tremeGene siRNA Transfection Reagent (Roche, Basel, Switzerland). At 36 hours post-transfection (hpt), the transfected cells were infected with PRRSV HuN4 at a MOI of 0.01. After 1 h, the cells were washed and incubated at 37 °C. At 24 and 48 hpi, the cell lysates were harvested for analysis by Western blotting. Viral RNA copy number and viral titers in the cell culture supernatants were also determined [26].

To construct a stable cell line overexpressing ANXA2, 293T cells grown in 10-cm cell dishes were co-transfected with 20 μg pFUGW-ANXA2 or pFUGW empty vector, together with 15 μg of pSPAX2 and 6 μg of pMD2G. The recombinant lentiviruses were harvested at 48 hpt and centrifugation at 6000 × *g* for 20 min at 4 °C using an Amicon Ultra-15 Centrifugal Filter Unit (Millipore, Burlington, MA, USA). The Marc-145 cells were then transduced with recombinant lentiviruses. At 36 hpt, the Marc-145 cells were infected with PRRSV HuN4 at a MOI of 0.01. At 48 hpi, cell culture supernatants and cell lysates were harvested for analysis.

### Construction of the vimentin, ANXA2, and truncated-ANXA2 mutant plasmids

The full ANXA2 and vimentin gene sequences were amplified and cloned into an empty pCMV-HA vector (Clontech, Mountain View, CA, USA) to generate pANXA2-Flag and pVIM-HA, respectively. In order to identify the binding domain of ANXA2, various domain positions in ANXA2 were analyzed. According to the positions of the four putative domains, five truncated ANXA2 fragments were designed and amplified by PCR. The primers for amplifying these fragments are listed in Table 1.

**Table 1:** **Primers used for expression plasmid construction**

Primers	Sequences (5′ to 3′)
pFU-ANXA2-F	CGCGGATCC ATGTCTACCGTTCATGAAATTCT
pFU-ANXA2-R	TAACACCGGTTCAGTCATCCCCACCACACAG
pANXA2-F	TTGGGCCCACCATGTCTACCGTTCATGAAATTCT
pANXA2-Flag-R	CCGCTCGAGTCACTTATCGTCGTCATCCTTGTAATCGTCATCCCCACCACACAG
pVIM-F	TTGGGCCCACCATGACCACCAGGTCCGTGTC
pVIM-HA-R	CCGGAATTCTTAAGCGTAATCTGGAACATCGTATGGGTATTCAAGGTCATCGTGATGC
ANXA2-Flag(1–102)-F	TTGGGCCCACCATGTCTACCGTTCATGAAATTCT
ANXA2-Flag(1–102)-R	CCGCTCGAGTCACTTATCGTCGTCATCCTTGTAATCTAGGCCCAAAATCACTGTC
ANXA2-Flag(1–174)-F	TTGGGCCCACCATGTCTACCGTTCATGAAATTCT
ANXA2-Flag(1–174)-R	CCGCTCGAGTCACTTATCGTCGTCATCCTTGTAATCCAGGGCAACCATCAGCTTG
ANXA2-Flag(1–259)-F	TTGGGCCCACCATGTCTACCGTTCATGAAATTCT
ANXA2-Flag(1–259)-R	CCGCTCGAGTCACTTATCGTCGTCATCCTTGTAATCCAGGTTCAGGAAAGCATTTT
ANXA2-Flag(109–340)-F	TTGGGCCCACCATGTATGACGCTTCCGAGCTGAAA
ANXA2-Flag(109–340)-R	CCGCTCGAGTCACTTATCGTCGTCATCCTTGTAATCGTCATCCCCACCACACAG
ANXA2-Flag(192–340)-F	TTGGGCCCACCATGCAAGATGCCCGGGATCTCTAT
ANXA2-Flag(192–340)-R	CCGCTCGAGTCACTTATCGTCGTCATCCTTGTAATCGTCATCCCCACCACACAG

### Confocal microscopy

PAMs and Marc-145 cells at 70% confluence were washed three times and then fixed with 4% paraformaldehyde at room temperature for 30 min, blocked with 5% skimmed milk for 2 h at room temperature, and then incubated with the respective antibodies and then examined using a Leica SP2 confocal system (Leica Microsystems, Wetzlar, Germany).

### Co-immunoprecipitation (Co-IP) and Western blotting

PAMs and Marc-145 cells were grown on 10-cm dishes and then harvested and washed three times with cold PBS. The cells were then lysed with 1% Triton X-100 containing protease inhibitor cocktail (Roche) on ice for 1 h. The cell lysates were then centrifuged at 13 000 × *g* for 10 min at 4 °C. The supernatants were precleared with Protein A/G PLUS-Agarose (Santa Cruz Biotechnology, Dallas, TX, USA) at 4 °C for 2 h and then incubated with anti-ANXA2 mAb with rotation at 4 °C for 2 h. The immunoprecipitates were washed five times with 1% Triton X-100 and bound proteins were analyzed by Western blotting.

Co-IP assays were also performed using exogenous ANXA2 or vimentin transfected 293T cells. The 293T cells were co-transfected with pVIM-HA and pANXA2-Flag, or with pVIM-HA and plasmids expressing the ANXA2 mutants (8 μg each), using X-tremeGENE HP DNA Transfection Reagent (Roche). At 48 hpt, the transfected cells were harvested, and lysed with 1% Triton X-100 containing protease inhibitor cocktail on ice for 1 h. Finally, the cell lysate was centrifuged at 13 000 ×* g* for 10 min at 4 °C. The supernatants were transferred to a tube containing equilibrated anti-HA affinity gel beads (Sigma-Aldrich) or anti-Flag affinity gel beads (Sigma-Aldrich) and vortexed briefly and incubated with gentle mixing for 2 h at 4 °C. In the experiment of ANXA2 is required for vimentin binding to N, the supernatants obtained from HEK 293T transfected with plasmids expressing tagged VIM and ANXA2 proteins alternatively mixed with PRRSV proteins for 2 h at 4 °C and then transferred to a tube containing equilibrated anti-Flag affinity gel beads. The mixtures were then vortexed briefly and incubated with gentle mixing for 2 h at 4 °C. Finally, the immunoprecipitates were washed five times and then analyzed by immunoblotting.

### Preparation of PRRSV proteins

Marc-145 cells were infected with PRRSV HuN4 (MOI = 0.01), and the cell cultures were harvested at 84 hpi. After freezing and thawing three times, the cellular debris was removed by centrifugation at 8000 × *g* for 10 min at 4 °C. The supernatant was centrifuged at 111 000 × *g* for 2 h at 4 °C, and the pellets were resuspended in PBS. Subsequently, the PRRSV proteins were extracted using mild extraction buffer 1% Triton X-100 containing protease inhibitor cocktail.

### Real-time reverse transcriptase PCR

Total RNA from PRRSV-infected cells was extracted using a QIAamp Viral RNA Mini Kit (Qiagen, Hilden, Germany) and then reverse transcribed with a random primer RT kit (TaKaRa, Kusatsu, Japan). The products were used as a template for quantifying PRRSV RNA copy number using a previously validated TaqMan fluorescent quantitative PCR method [26].

### Virus titration

Marc-145 cells, grown in 96-well plates, were infected with tenfold serial dilutions of PRRSV HuN4. After 1 h incubation at 37 °C, the supernatants were replaced with fresh DMEM containing 2% FBS. Viral titers were determined using endpoint dilution analysis at 5 dpi. The Reed–Muench method was used to determine the 50% tissue culture infected dose (TCID_50_) [27].

### Statistical analysis

Data are expressed as mean ± standard deviation (SD). Differences between groups were examined for statistical significance using Student’s *t* tests. An unadjusted *P* value of < 0.05 was considered statistically significant.

## Results

### ANXA2 expression is higher in PAMs infected with PRRSV HuN4

**Figure 1:**
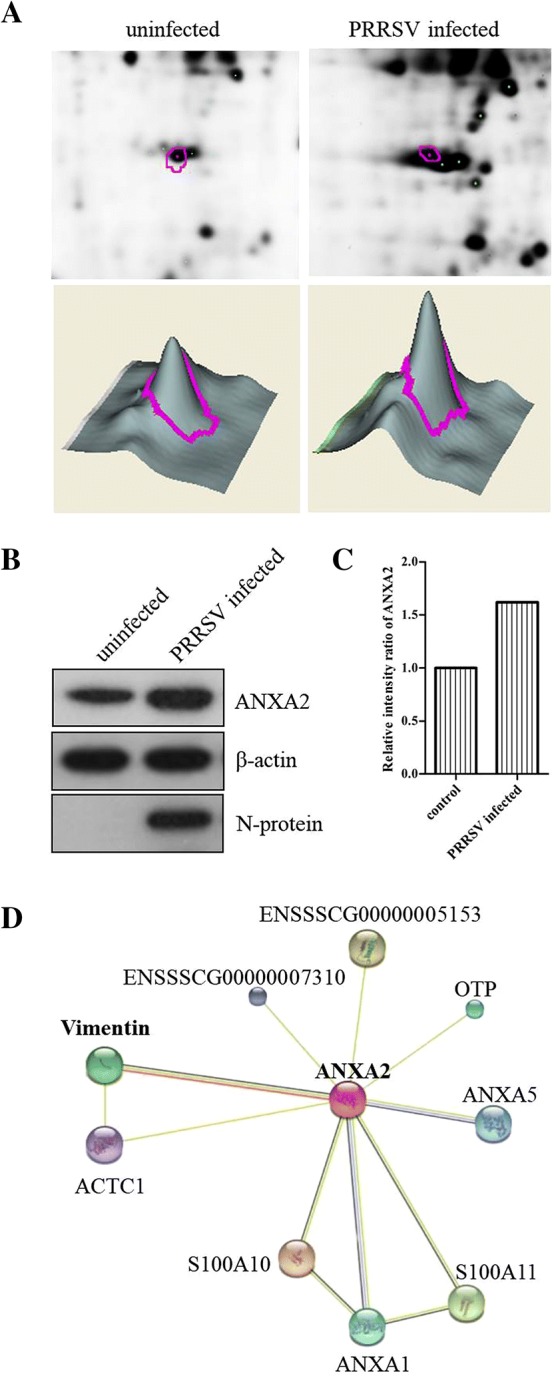
**ANXA2 was identified as a differentially upregulated protein by 2D-DIGE/MS analysis. A** PAMs were infected with PRRSV HuN4 at a MOI of 0.01. At 48 hpi, cells were harvested and analyzed by 2D-DIGE. The 2D-DIGE profiles of differentially expressed protein levels in either the uninfected control PAM or HuN4-infected PAM. The 3D spot intensity represents the differentially expressed ANXA2 protein. **B** ANXA2 was identified using Western blotting. PAMs were infected with PRRSV HuN4 at a MOI of 0.01. At 48 hpi, cells were harvested for analysis by Western blotting. **C** The relative intensity ratios of ANXA2. **D** Bioinformatics analysis about ANXA2 was conducted using STRING and the interaction network revealed that ANXA2 and other proteins (including vimentin) form a protein interaction network.

Proteins from infected and uninfected cells were separated using 2D-DIGE. Using an independent two-way ANOVA, 15 of the most significant spots were selected and sent for identification using MALDI-TOF/TOF mass spectrometry. Fourteen of the spots were successfully identified, with 11 of the 14 matching their homologous protein in databases (Table 2). Of these 11 differentially expressed proteins, ANXA2 was found to be upregulated when compared with uninfected PAM cells (Figures 1A–C). Subsequent bioinformatics analysis indicated that ANXA2 was potentially involved in a protein interaction network that included several other proteins, including vimentin (Figure 1D). The results indicated that ANXA2 is a differential protein and may be relevant to vimentin.

**Table 2:** **MALDI-TOF/TOF mass spectrometry of the selected differential expressed proteins of PRRSV-infected PAM cells**

Protein ID	Protein name	Accession no.	Protein MW	Protein PI	Protein score	Protein score C%	Peptide count	Up (↑) or down (↓) regulation
1137	Plasma gelsolin precursor	gi|164472	85 064.9	5.93	255	100	16	↑
1629	Annexin A4	gi|264681432	36 034.2	5.71	769	100	26	↑
1664	Cathepsin B precursor	gi|147906534	37 902.9	5.81	286	100	12	↑
1794	Lectin galactoside-binding soluble 3	gi|124830340	27 311.5	8.78	387	100	13	↓
1845	F-actin capping protein beta subunit	gi|148613359	30 951.5	5.69	179	100	15	↓
2000	Proteasome subunit alpha type 6	gi|210062872	27 884	6.34	178	100	10	↑
2188	Similar to peroxiredoxin 4	gi|194044822	30 764	6.01	212	100	4	↓
2363	Annexin 1	gi|13399614	39 005.2	6.37	167	100	10	↑
2385	Annexin A2	gi|54020966	38 794.8	6.49	681	100	18	↑
2707	Calcium-binding protein A9	gi|226442051	15 996.7	6.41	83	99.9	7	↓
2839	Galectin-1	gi|47716872	14 932.3	5.07	444	100	9	↑

Fifteen of the most significant spots were selected and sent for identification by using MALDI-TOF/TOF mass spectrometry. Fourteen of them the spots were successfully identified, by mass spectrometry and, with 11 of the 14 proteins were matched for their homologous protein.

### ANXA2 is critical for PRRSV replication

**Figure 2:**
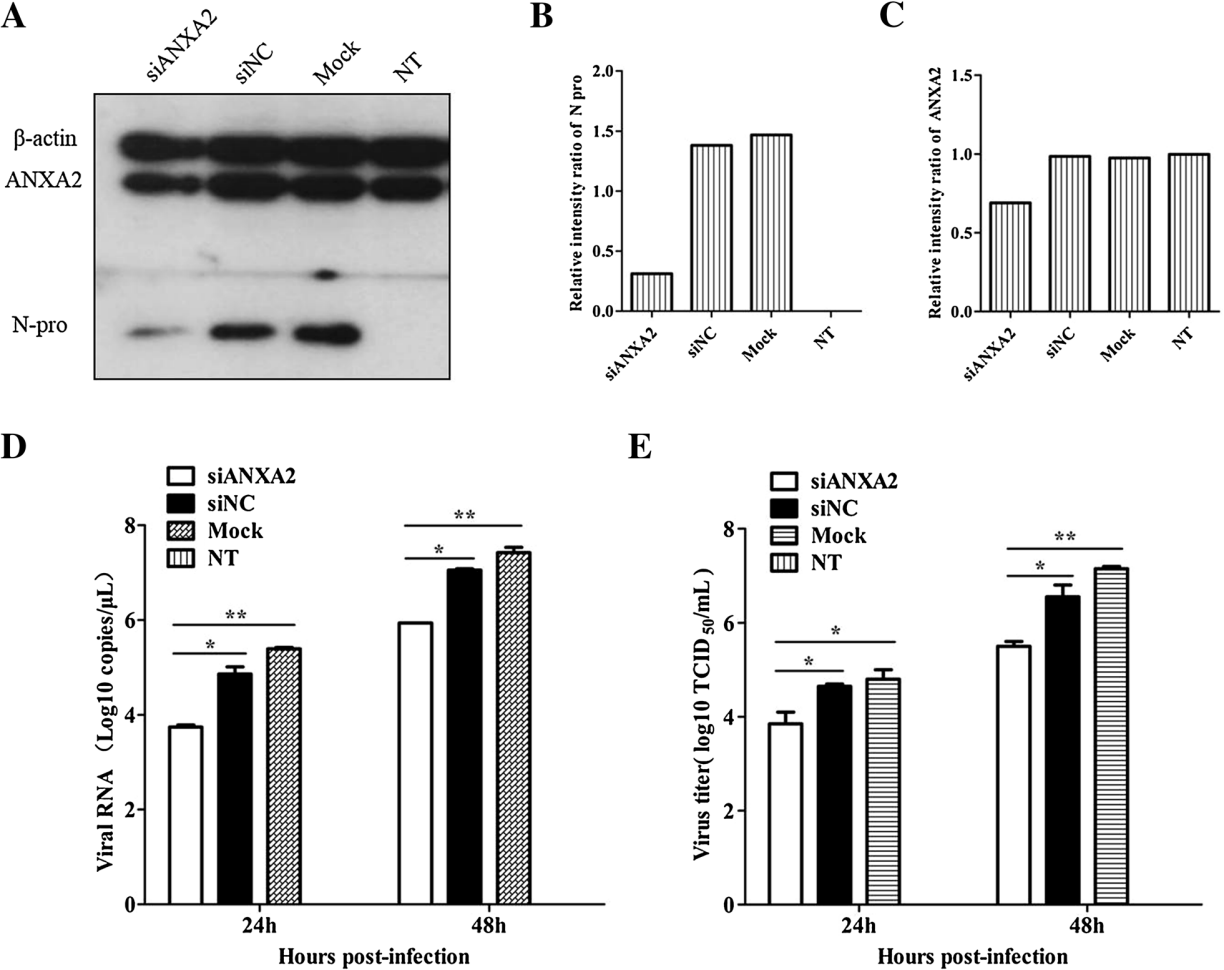
**ANXA2 knockdown by siRNA and its effect on PRRSV multiplication. A** Reduced ANXA2 expression by siRNA. Marc-145 cells transfected with 100 nM siANXA2 or a non-targeting control (siNC), transfection reagent (mock treatment), or left untreated (no treatment; NT) for 36 h were infected with PRRSV HuN4 at a MOI of 0.01 for 24 and 48 h, and then collected the samples for Western blotting using mouse anti-β actin monoclonal antibody (GenScript), rabbit anti-ANXA2 monoclonal antibody (Cell Signaling Technology) and mouse anti-PRRSV N protein monoclonal antibody. **B** The relative intensity ratios of N protein and **C** ANXA2 in a Western blot showing successful knockdown. **D** PRRSV replication in ANXA2 knockdown cells obtained from cell culture supernatants treated with PRRSV HuN4 was assessed by RNA copy number by quantitative real-time RT-PCR assay and **E** PRRSV protein titers. *P < 0.05; **P < 0.01.

Specific siRNAs were used to down-regulate the expression of ANXA2 in Marc-145 cells. Western blotting confirmed that expression of ANXA2 and the PRRSV N protein were decreased relative to cells treated with a non-targeting siNC, transfection reagent alone (mock treated), and untreated (no treatment; NT) controls at 48 hpi (Figures 2A–C). In addition, viral genome copy numbers in the ANXA2 knockdown cells decreased at 24 and 48 hpi relative to the controls (Figure 2D). Similarly, PRRSV titers decreased after ANXA2 knockdown in the Marc-145 cells (Figure 2E). Together, these results show that knockdown of ANXA2 negatively modulated PRRSV reproduction. In comparison, Western blotting of overexpressing ANXA2 in Marc-145 cells demonstrated that the abundance of the protein had increased, although N protein expression levels and PRRSV genome copy number remained unchanged when compared to the controls (data not shown).

### Confocal analysis of ANXA2 and vimentin

**Figure 3:**
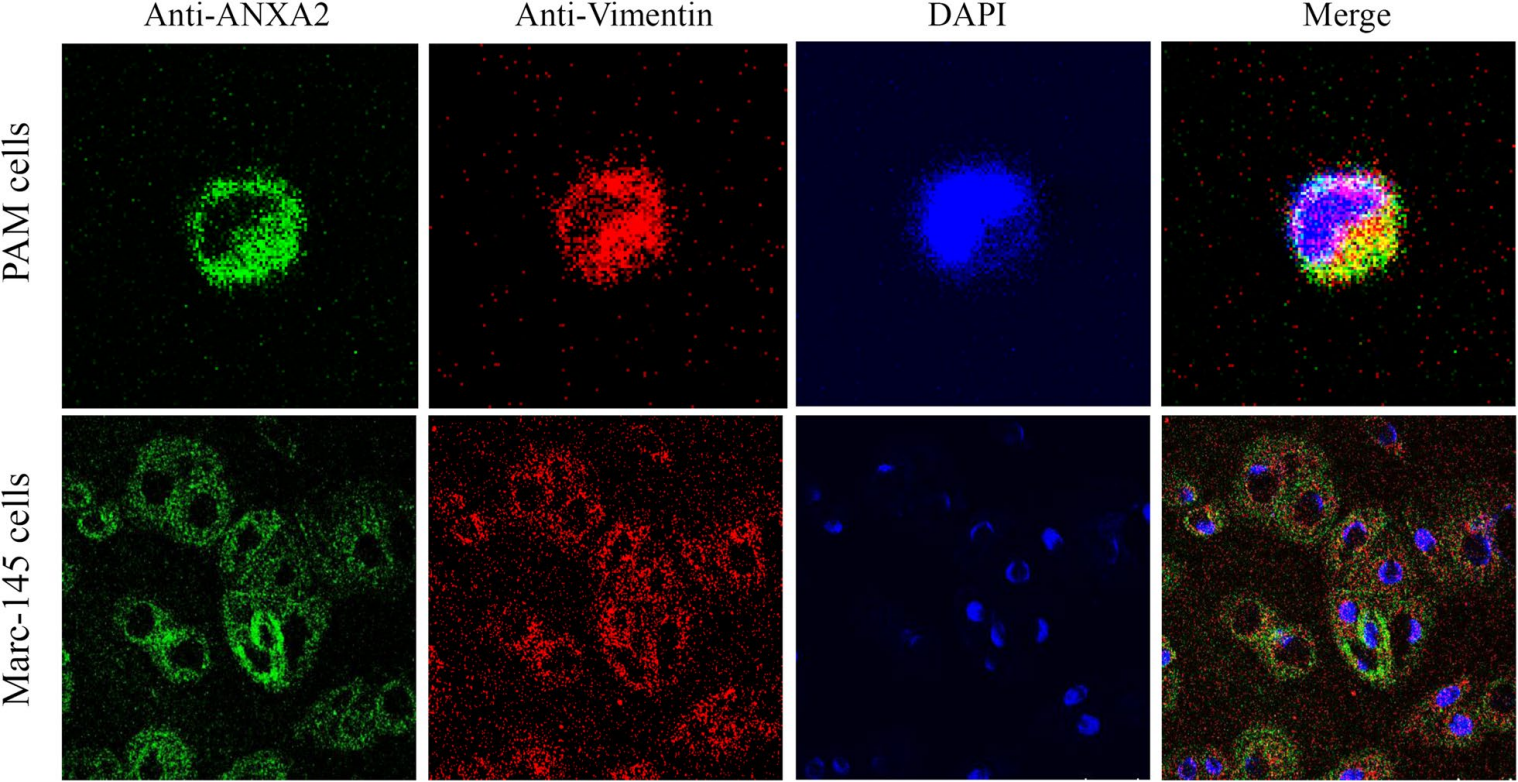
**Co-localization of ANXA2 and vimentin in PAMs, Marc-145 cells.** PAMs and Marc-145 cells at 70% confluence were washed, fixed, blocked with 5% skimmed milk and then incubated with rabbit anti-ANXA2 mAb (Cell Signaling Technology) or mouse anti-vimentin mAb (Cell Signaling Technology) at 4 °C overnight and then incubated for 1 h with goat anti-rabbit IgG-FITC antibodies or goat anti-mouse IgG-TRITC antibodies. Finally, the cells were then counter-stained with DAPI and subjected to confocal assay. Fluorescent staining of ANXA2 (green) and vimentin (red) in PAMs and Marc-145 cells, counterstained with DAPI (blue). The scale bar indicates 10 µm.

Bioinformatics analysis suggested that ANXA2 may interact with vimentin in an interactomic network. To determine if ANXA2 interacts as a partner of vimentin, confocal analysis was performed using PAMs and Marc-145 cells to reveal that ANXA2 co-localized with vimentin (Figure 3), suggesting an interaction. In addition, in ANXA2-Flag and vimentin-HA co-transfected 293T cells, co-localization was also observed (data not shown).

### Co-immunoprecipitation (Co-IP) of ANXA2 and vimentin

**Figure 4:**
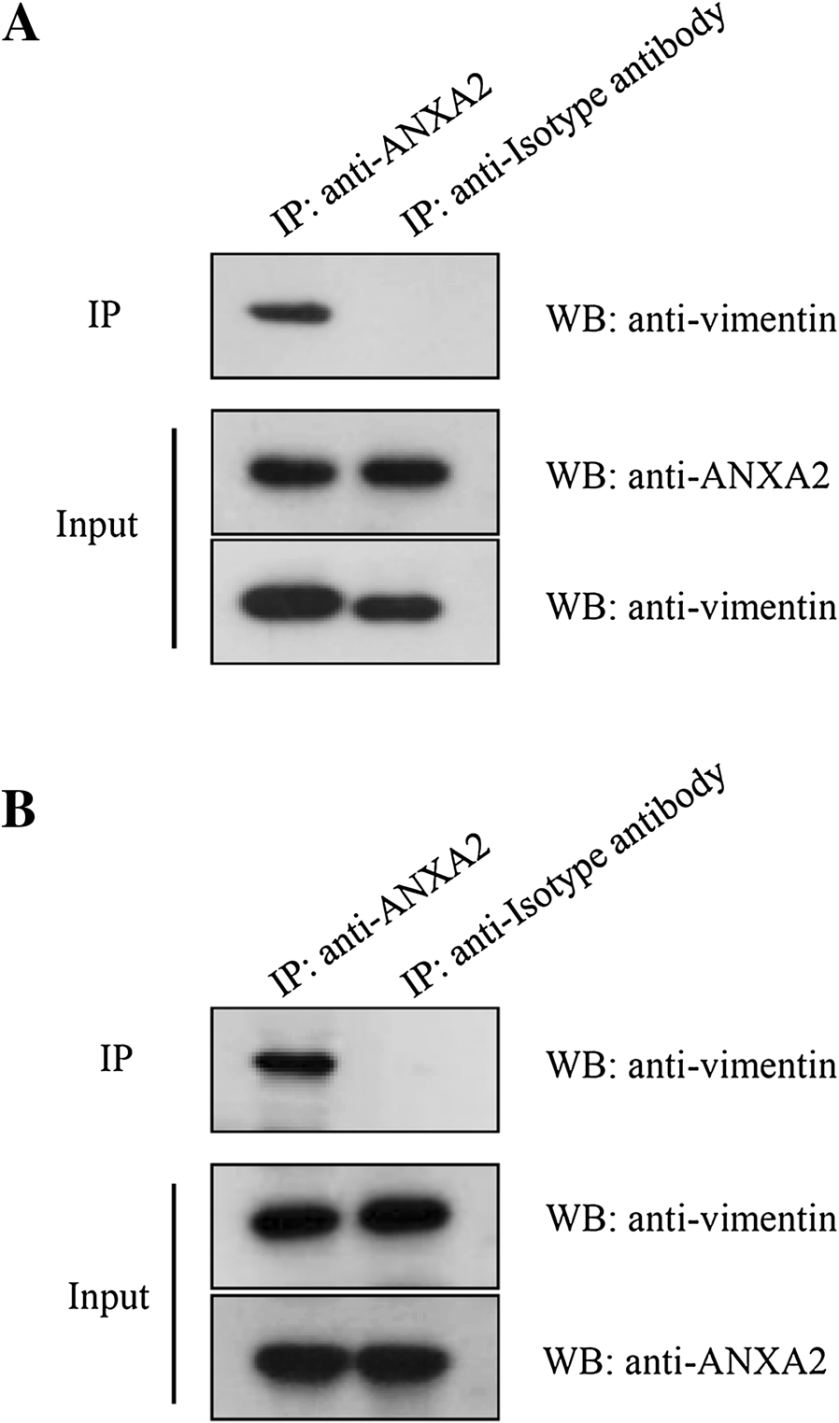
**ANXA2 interacts with vimentin.** PAMs (**A**) and Marc-145 cells (**B**) were harvested and subjected to co-IP assay using anti-ANXA2 monoclonal antibody and the precipitated proteins were analyzed by Western blotting using antibodies against ANXA2 and vimentin proteins.

**Figure 5:**
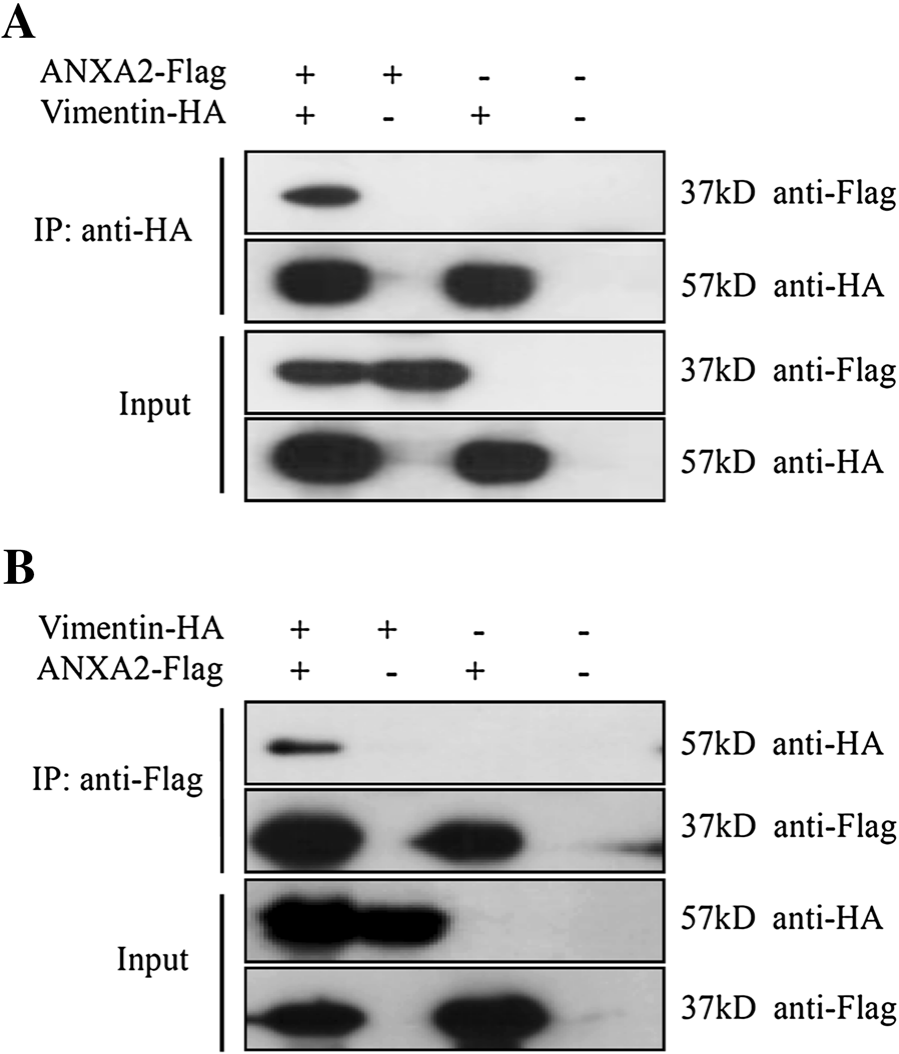
**Interaction between ANXA2-Flag and vimentin-HA in 293T cells.** 293T cells co-transfected with pCMV-ANXA2-Flag and pCMV-vimentin-HA were harvested at 48 h and subjected to co-IP assay using mouse **A** anti-HA affinity gel or **B** anti-Flag affinity gel using. The precipitated proteins were analyzed by Western blotting using antibodies against Flag or HA.

To further confirm that an interaction existed between ANXA2 and vimentin, endogenous Co-IP assays were performed using PAMs and Marc-145 cells. The results showed that intracellular ANXA2 precipitated with endogenous vimentin in both PAMs and Marc-145 cells (Figure 4). In a subsequent exogenous Co-IP assay, ANXA2-Flag and vimentin-HA proteins expressed in 293T cells were employed. And the results revealed that exogenous ANXA2-Flag and vimentin-HA also could specifically interact with either vimentin-HA or ANXA2-Flag, respectively (Figure 5). Together, these results confirmed that ANXA2 is a binding partner of vimentin.

### The B domain of ANXA2 is required for the ANXA2-vimentin interaction

**Figure 6:**
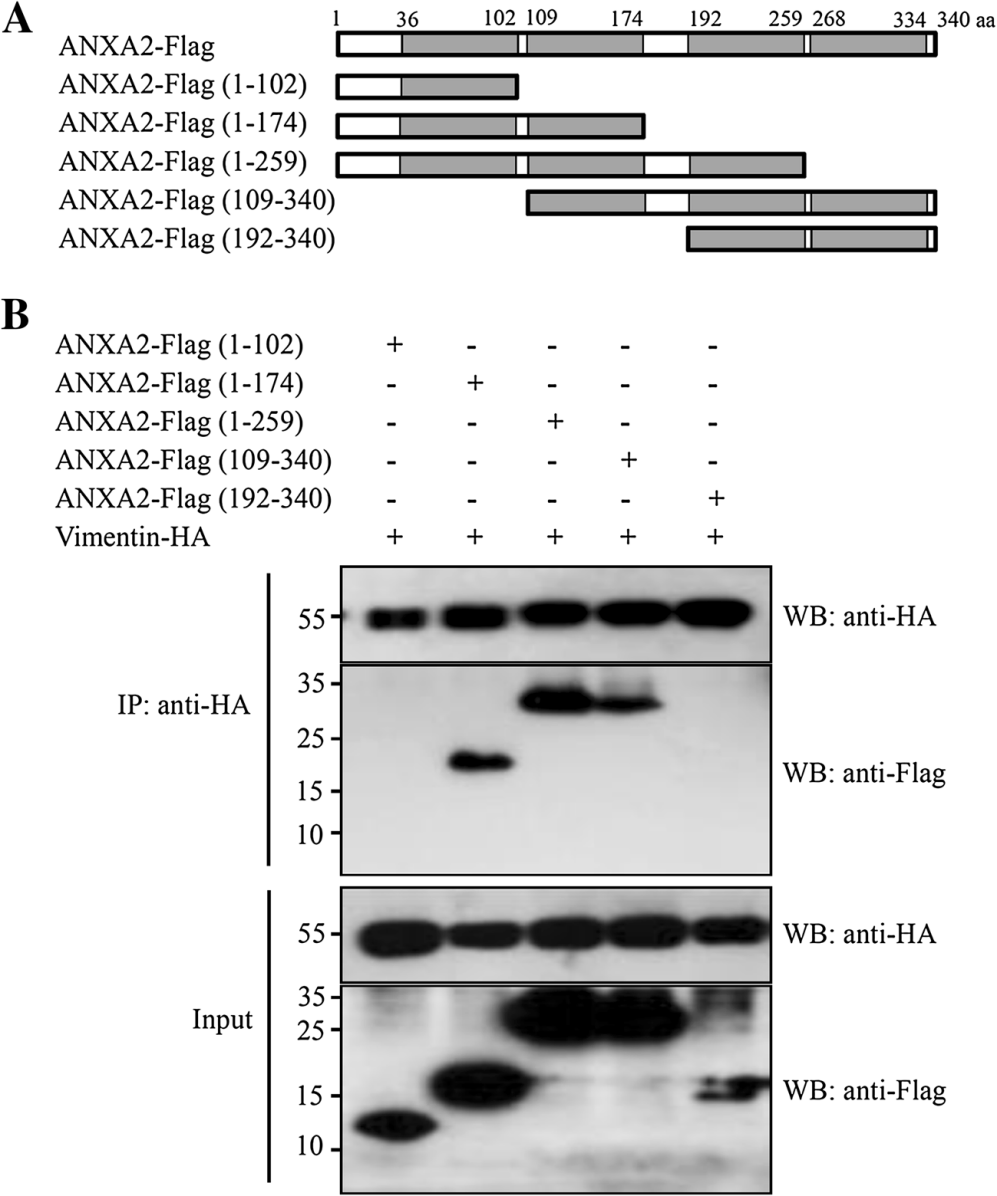
**Interaction between vimentin and a series of ANXA2-Flag mutants. A** Schematic representation of the predicted protein domains of ANXA2. The full-length ANXA2 protein and five truncated ANXA2 mutants were examined in this study. **B** 293T cells co-transfected with pVIM-HA and plasmids expressing the ANXA2 mutants were collected at 48 h and subjected to co-IP assay using mouse anti-HA affinity gel. The precipitated proteins were analyzed by Western blotting using antibodies against Flag or HA.

To map the region of ANXA2 required for vimentin binding, five plasmids expressing various Flag-tagged truncated ANXA2 mutants were constructed (Figure 6A). These plasmids were co-transfected with HA-tagged vimentin into HEK293T cells. Further Co-IP assays showed that vimentin interacted with ANXA2-Flag (1–174 aa), ANXA2-Flag (1–259 aa), and ANXA2-Flag (109–340 aa), but not with ANXA2-Flag (1–102 aa) nor ANXA2-Flag (192–340 aa) (Figure 6B). Finally, we attempted to directly establish whether the B domain alone could bind the ANXA2-vimentin complex. However, we were unable to detect the antigen–antibody complex on Western blots due to the small molecular weight of the fragment (7 kDa) and low expression levels (data not shown). Despite this, these findings indicate that the B domain (109–174 aa) of ANXA2 is required for the interaction between vimentin and ANXA2.

### ANXA2 is required for vimentin binding to the PRRSV N protein

**Figure 7:**
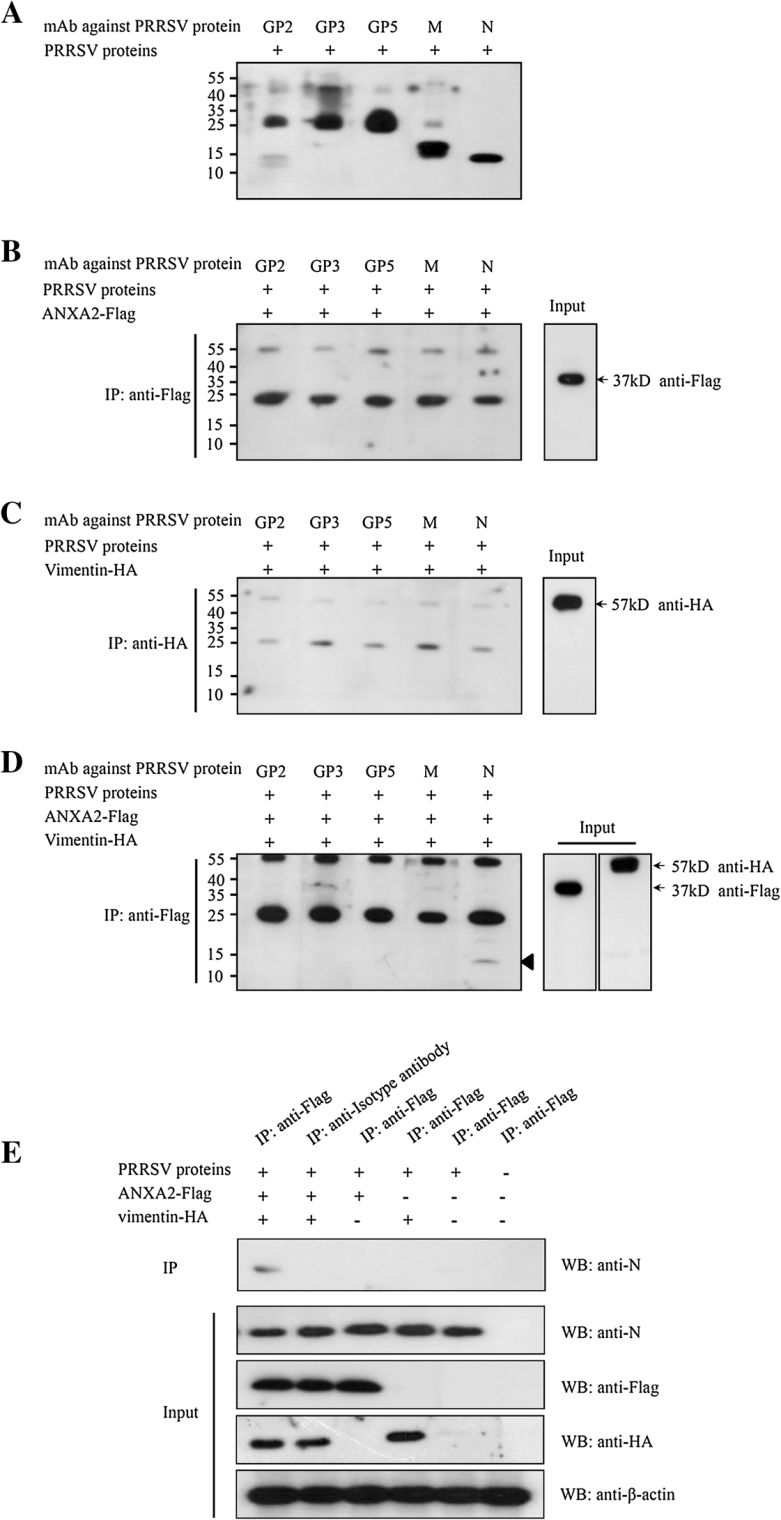
**ANXA2/vimentin binding to PRRSV structural proteins. A** Validation of the reactivity of PRRSV structural protein mAbs and the PRRSV proteins by SDS-PAGE. **B** The mixture of PRRSV protein incubated with the extraction of HEK293T transfected with plasmids expressing tagged ANXA2 protein and subjected to Co-IP. The precipitated proteins were performed using anti-GP2, anti-GP3, anti-GP5, anti-M, and anti-N mAbs. **C** The binding of vimentin to PRRSV structural proteins. The mixture of PRRSV protein incubated with the extraction of HEK293T transfected with plasmids expressing tagged vimentin protein and then subjected to Co-IP. **D** The binding of the ANXA2-vimentin complex to PRRSV structural proteins. The mixture of PRRSV protein mixed with ANXA2-vimentin complex, and then performed the Co-IP assay. The triangle (black up-pointing triangle) highlights the position of the PRRSV N protein. **E** The mixture of PRRSV protein mixed with HEK293T transfected with plasmids expressing tagged ANXA2 and vimentin protein or control vector, and then performed Co-IP assay using anti-Flag affinity gel. The precipitated proteins were tested by Western blotting using the corresponding antibodies.

To investigate the role that ANXA2 plays in the binding of vimentin to PRRSV, 293T cells were transfected individually or simultaneously with pVIM-HA and pANXA2-Flag. Next, the expressed protein(s) were allowed to bind to PRRSV proteins and then probed with a series of mAbs specific to various viral proteins (anti-GP2, anti-GP3, anti-GP5, anti-M protein, and anti-N protein). This showed that, while of all the mAbs recognized their corresponding PRRSV proteins (Figure 7A). In addition, ANXA2 and vimentin expressed individually did not interact with GP2, GP3, GP5, nor the M or N proteins of PRRSV (Figures 7B, C). Only when ANXA2 and vimentin exist together could interact with the N protein. No interaction was found between vimentin and GP2, GP3, GP5, or the M protein of PRRSV, even when expressed in tandem with ANXA2 (Figures 7D, E). These findings suggest that ANXA2 is an important co-factor involved in the binding of vimentin to the PRRSV N protein.

## Discussion

Here, we have shown that ANXA2 can promote the replication of PRRSV. Moreover, ANXA2 was confirmed that could interact with vimentin for the first time. In addition, the ANXA2-vimentin complex has the ability to bind to the PRRSV N protein. Individually, neither ANXA2 nor vimentin can bind to PRRSV, suggesting that ANXA2 is a necessary accessory molecule for vimentin binding. Since the N protein isn’t exposed on the surface of PRRS virion, the ANXA2 may not be involved in the entry process, and ANXA2/vimentin interaction may be important cellular factors for PRRSV multiplication. The interaction between NSP2 and vimentin and N protein was well confirmed [19, 20]. Stable overexpression of ANXA2 in Marc-145 cells did not enhance the infectivity nor multiplication of PRRSV. Although this supports data from a previous study [25], the reasons for this are unclear and may be related to endogenous levels of vimentin.

Supporting our study, both ANXA2 and vimentin have been shown to play a role in viral disease, although ours is the first to demonstrate that the two proteins interact. Vimentin has previously been demonstrated to act as an attachment receptor for enterovirus 71 [28] and can also interact with dengue virus nonstructural protein 4A, a component of the viral replication complex [29]. ANXA2 is a member of the annexin superfamily of proteins that are calcium-dependent phospholipid binding proteins responsible for mediating essential several biological processes, including membrane trafficking, endosome formation, and vesicle aggregation [16, 30]. Consequently, ANXA2 itself is found in a wide variety of cells and tissues and is a key host factor involved in many diverse cellular processes, including vesicle trafficking [31], membrane fusion [32], and exocytosis [33]. ANXA2 has been found to be involved in various viral infections, including cytomegalovirus [34], hepatitis C virus [17], and infectious bursal disease virus [35]. ANXA2 also has an important role during human immunodeficiency virus (HIV) type 1 entry, assembly, and budding [36]. In classical swine fever virus infections, ANXA2 has been shown to be involved in several events, such as viral attachment and entry, replication, assembly, and the budding and release of viral particles from host cells [37–39]. ANXA2 is also a potential receptor for respiratory syncytial virus in human epithelial cells [40]. Here, we have shown that ANXA2 is an important cellular factor for vimentin and PRRSV replication.

Vimentin has also been identified as an important part of the PRRSV receptor complex [19, 41, 42]. Notably, it has been reported that vimentin indirectly interacts with nonstructural protein 2 (NSP2) of PRRSV in the presence of N protein [20] and experiments using vimentin bound to PRRSV N protein and anti-vimentin antibodies showed that PRRSV activity could be blocked [19]. Additionally, the interaction between the N protein, NSP9, and cellular DHX9 was found to regulate viral RNA synthesis [43] and NSP2 has been shown to form a complex with vimentin using the N protein as an intermediate [20]. A limitation of our study is that interactions between ANXA2-vimentin and GP4, and various NSP proteins, could not be examined due to the lack of available mAbs. Further investigation is therefore required to establish if there is binding between ANXA2, vimentin, and either GP4 or NSPs, in addition to binding to the N protein.

In conclusion, our data show for the first time that ANXA2 is an essential molecule for the binding of vimentin and that the ANXA2 B domain of contributes to this interaction. Furthermore, the ANXA2-vimentin complex has the ability to bind the PRRSV N protein. Our work provides important information concerning the regulation of PRRSV replication and may lead to better control efforts to manage this economically important disease.

## Abbreviations

PRRSV: porcine reproductive and respiratory syndrome virus
ANXA2: annexin A2
PAMs: porcine alveolar macrophages
ORFs: open reading frames
PBS: phosphate-buffered saline
DMEM: Dulbecco’s modified Eagle’s medium
FBS: fetal bovine serum
2D-DIGE: two dimension difference gel electrophoresis
hpi: hours post-infection
IEF: isoelectric focusing
TCID_50_: 50% tissue culture infected dose
