# Correction to: Annexin A2 binds to vimentin and contributes to porcine reproductive and respiratory syndrome virus multiplication

**DOI:** 10.1186/s13567-018-0595-x

**Published:** 2018-10-05

**Authors:** Xiao‑Bo Chang, Yong‑Qian Yang, Jia‑Cong Gao, Kuan Zhao, Jin‑Chao Guo, Chao Ye, Cheng‑Gang Jiang, Zhi‑Jun Tian, Xue‑Hui Cai, Guang‑Zhi Tong, Tong‑Qing An

**Affiliations:** 1 State Key Laboratory of Veterinary Biotechnology, Harbin Veterinary Research Institute, Chinese Academy of Agricultural Sciences, Harbin, 150069 China; 2 Shanghai Veterinary Research Institute, Chinese Academy of Agricultural Sciences, Shanghai, 200241 China

## Correction to: Vet Res (2018) 49:75 10.1186/s13567-018-0571-5

In the original publication of this article [1], the author found the brand of vimentin antibody was wrong in Figure 3. The legend of Figure 3, “mouse anti-vimentin mAb (Cell Signaling Technology) at 4 °C overnight” should be “mouse anti-vimentin mAb (Sigma-Aldrich) at 4 °C overnight”.
